# Introduction to the special issue on Down syndrome

**DOI:** 10.1590/1980-5764-DN-2025-E002

**Published:** 2026-03-23

**Authors:** Mônica Sanches Yassuda

**Affiliations:** 1 Universidade de São Paulo, São Paulo SP, Brazil.

 In 2025, the editors of the journal Dementia & Neuropsychologia, dedicated to the field of neurosciences, with an emphasis on cognition and behavior, elected the topic of Down syndrome (DS) for the organization of a special issue. This decision was supported by the growing interest in DS, associated, among other reasons, with the greater longevity of individuals with the syndrome. Studies with people with DS over 35 years of age have contributed significantly to the understanding of the biological mechanisms of neurodegenerative diseases such as Alzheimer’s disease (AD). 

 Recent research indicates that individuals with DS have a genetic form of AD, as they possess an additional copy of the APP gene, due to the trisomy of chromosome 21. The longevity of individuals with DS has shown that almost all develop the neuropathology of AD around the age of 40^1^. It is estimated that 70% have dementia around age 54, with the lifetime risk for AD reaching 95%^2^. Furthermore, AD is the leading cause of death after age 35 among people with DS^3^. In this segment of the population, AD pathology evolves from intraneuronal beta-amyloid in childhood. Later, diffuse plaques are found in adolescence and early adulthood, and there is significant presence of fibrillar beta-amyloid and neurodegeneration around age 40^4^. Neuroinflammation intensifies throughout the life cycle, and cerebral amyloid angiopathy can be observed around age 55^5^. The faster progression of AD in people with DS offers opportunities for new insights into the disease and opportunities for testing new drugs^4^. 

 Considering these relatively recent findings, the editors of the journal Dementia & Neuropsychologia deemed it opportune to open a call for papers for a special issue dedicated to DS. Below, we highlight the topics covered in the approved articles. 

 Natividade et al.^6^, in "Causes of Down syndrome regression disorder: a scoping review", mapped the available evidence regarding the possible causes of Down Syndrome Regression Disorder (DSRD) and the factors that may contribute to its development. It is an acute or subacute condition of neurocognitive regression that impacts the autonomy and quality of life of people with DS. The identified causes converge on a neuroinflammatory process and psychosocial stressors, which may act as triggers. 

 Silva et al.^7^, in "Genetic markers involved in neuroinflammation in Down syndrome: a systematic review", sought to identify genetic markers involved in neuroinflammatory processes in individuals with DS. In all, 63 genes and 42 genetic markers associated with neuroinflammation in DS were identified. These genes showed variations in expression that may alter inflammatory responses, indicating a possible relationship with the progression of neurodegenerative diseases in this population. 

 Fonseca et al.^8^, in "Inclusive research with individuals with Down syndrome at risk for dementia", defined inclusive research as a partnership between academic researchers and individuals who are experiencing the investigated condition. Inclusive research on intellectual disability and risk of dementia is an area that is under-explored and under-documented. The authors described the evolution of inclusive research in this area and presented some of the challenges and benefits of its implementation, as well as the main aspects to be considered in planning such studies. 

 Carvalho et al.^9^, in "Assessment of language abilities and functionality in adults and elders with Down syndrome", evaluated linguistic abilities and functionality in people with DS. The Arizona Battery for Communication Disorders in Dementia (ABCD), Object and Action Naming Battery (OANB) and Lawton & Brody Activities of Daily Living (ADL) scales were used for linguistic and functional assessment. The literate group showed better functional performance in all subdomains of the ABCD (except for Linguistic Expression) and the OANB. There was an association between the Word Learning, Repetition, Generative Naming tests and functional status in the literate group. In the non-literate group, Word Learning, Object Description, Comparative Questions and Repetition were associated with the total functional score. 

 Nascimento Filho et al.^10^, in "Current evidence on drug therapy for dementia in people with Down syndrome: an overview of systematic reviews", assessed the effectiveness of pharmacological interventions to treat dementia in individuals with DS. Five systematic reviews were included and they analyzed the effects of simvastatin, antioxidants, acetyl-L-carnitine, anticonvulsants, acetylcholinesterase inhibitors (donepezil), NMDA receptor antagonists (memantine), and rapid-acting intranasal insulin on dementia symptoms in patients with DS. All reviews indicated low levels of evidence for the efficacy of the drugs mentioned above. 

 Carvalho et al.^11^, in "Object and action naming in adults and aged people with Down syndrome", evaluated changes in naming in adults and elderly with DS using the OANB. They also examined the influence of age, education and degree of intellectual disability (ID) on linguistic performance. Education had a greater impact on linguistic performance than the degree of ID or age. The OANB proved to be a useful tool for identifying performance patterns and supporting interventions for populations with DS. 

 Gomes et al.^12^, in "HSPA13 gene and microRNA-155: relationship between Down syndrome and Alzheimer’s disease", investigated in silico the differentially expressed genes (DEGs) that encode heat shock proteins and their interactions with microRNAs (miRNAs) located on human chromosome 21 (Hsa21). The results indicated that certain genes encoding members of the HSP family, particularly the interaction between miR155-5p and HSPA13, may be associated with AD in DS. 

 Nascimento et al.^13^, in "Assessment of cognitive function in individuals with Down Syndrome and Dementia: A systematic review" conducted a systematic review to examine the main cognitive changes in people with dementia in DS and the instruments used. After reviewing 28 studies, they concluded that, in early-stage dementia in DS, a significant decline in executive functions, memory, language, attention, and behavioral changes is observed. Among the behavioral symptoms, agitation, apathy, and hallucinations are common in AD-DS and prodromal dementia, and they can be considered as early markers. 

## Data Availability

The datasets generated and/or analyzed during the current study are available from the corresponding author upon reasonable request.
